# Bimodal Benefits for Lexical Tone Recognition: An Investigation on Mandarin-speaking Preschoolers with a Cochlear Implant and a Contralateral Hearing Aid

**DOI:** 10.3390/brainsci10040238

**Published:** 2020-04-17

**Authors:** Hao Zhang, Jing Zhang, Hongwei Ding, Yang Zhang

**Affiliations:** 1 Speech-Language-Hearing Center, School of Foreign Languages, Shanghai Jiao Tong University, Shanghai 200240, China; zhang.hao@sjtu.edu.cn (H.Z.); elainezhang@sjtu.edu.cn (J.Z.); 2 Department of Speech-Language-Hearing Sciences, University of Minnesota, Minneapolis, MN 55455, USA

**Keywords:** lexical tones, bimodal benefit, speech learning, cochlear implant (CI), hearing aid (HA)

## Abstract

Pitch perception is known to be difficult for individuals with cochlear implant (CI), and adding a hearing aid (HA) in the non-implanted ear is potentially beneficial. The current study aimed to investigate the bimodal benefit for lexical tone recognition in Mandarin-speaking preschoolers using a CI and an HA in opposite ears. The child participants were required to complete tone identification in quiet and in noise with CI + HA in comparison with CI alone. While the bimodal listeners showed confusion between Tone 2 and Tone 3 in recognition, the additional acoustic information from the contralateral HA alleviated confusion between these two tones in quiet. Moreover, significant improvement was demonstrated in the CI + HA condition over the CI alone condition in noise. The bimodal benefit for individual subjects could be predicted by the low-frequency hearing threshold of the non-implanted ear and the duration of bimodal use. The findings support the clinical practice to fit a contralateral HA in the non-implanted ear for the potential benefit in Mandarin tone recognition in CI children. The limitations call for further studies on auditory plasticity on an individual basis to gain insights on the contributing factors to the bimodal benefit or its absence.

## 1. Introduction

As a highlight of groundbreaking neural prosthesis in bioengineering, the cochlear implant (CI) provides accessibility to sound for individuals with profound hearing impairment [1,2]. The remarkable success of the CI is largely attributable to the proper use of signal transduction technology to harness central auditory plasticity of the implantees [2]. While CI users can significantly benefit from the neuroplasticity driven by the CI-empowered learning experience in developing their auditory, linguistic, and cognitive skills [3,4,5,6,7,8,9], pitch perception poses a unique challenge for these individuals. A vast body of literature demonstrated that CI recipients show deficits in pitch-related perceptual tasks, including voice emotion perception [10,11,12], speech prosody recognition [13,14,15], music appreciation [16,17,18], and lexical tone perception [19,20,21]. One known factor here lies in the limitations of the contemporary CI multichannel technology that encodes degraded spectral-temporal signals of the auditory input. Degradations in the representation can impair and even preclude the reception of pitch cues. The current study on Mandarin Chinese speakers aimed to investigate applicable interventions in alleviating the deficits in the representation and reception of pitch information for the pediatric CI users.

There has been a growing number of CI prescriptions not only for individuals with bilateral deafness but also for those with profound unilateral hearing loss. Some clinical practices point to compelling evidence in improving CI individuals’ speech perception by means of bimodal hearing for unilaterally implanted candidates with some residual acoustic hearing. Bimodal hearing refers to the combination of two different solutions for hearing loss, with fitting a CI in one ear while using a hearing aid (HA) in the opposite ear. Behaviorally, successful bimodal listeners obtained an improvement in speech understanding with CI + HA stimulation (i.e., a unilateral CI together with a contralateral HA) relative to with a unilateral CI alone [22,23,24]. The improved perceptual outcome in the bimodal stimulation over the CI alone condition is referred to as bimodal benefit, and it has been demonstrated in a number of studies of non-tonal languages. Several research groups provided corroborating evidence for bimodal benefits with task-specific tests in the perception of segmental linguistic features that contain low-frequency components, such as voicing, semivowels, and nasals [25,26], or in the perception of supra-segmental linguistic features, such as intonation, emphasis, and stress [27,28]. The behavioral improvements might be underpinned by the plasticity in auditory-related cortical areas as a consequence of the bimodal hearing experience. Nevertheless, the great heterogeneity of the CI population revealed a remarkable variability with respect to the degree of benefit from the bimodal stimulation. Some subjects tended to benefit significantly from the additional HA in the opposite ear, whereas others benefited less, or even not at all.

The great variability of the bimodal benefit leads to an initiative to pinpoint the contributing factors for this situation at the individual level. One important factor could be the degree of impairment in basic auditory function, which can lead to different amounts of residual hearing in the non-implanted ear [29]. For example, Most et al. [28] revealed significant negative correlations between the perception of supra-segmental features with bimodal stimulation and the unaided pure-tone average (PTA) of the non-implanted ear. However, it should be noted that the authors did not evaluate the relationship between the benefit magnitude in perceptual outcomes and the unaided PTA of the non-implanted ear directly. This might limit the power of validation on bimodal benefit for the individual participants. In another study, Zhang et al. [30] demonstrated that the amount of bimodal benefit in speech understanding was significantly correlated with audiometric thresholds of the non-implanted ear (r = −0.814). Nevertheless, some studies failed to find a reliable relationship between acoustic hearing thresholds and benefit magnitudes [31], which points to other possible contributing factors for bimodal benefits.

In recent years, there has been a surge in investigating the bimodal benefit on lexical tone perception in individuals from tonal languages [31,32,33,34,35,36]. Lexical tones are a distinctive feature of tonal languages, which utilize pitch variations to indicate contrasts in word meaning. For instance, Mandarin Chinese has four tones that can be combined with the same syllable to indicate different words (e.g., /ma/ could respectively mean ‘mother’, ‘hemp’, ‘horse’ or ‘scold’ depending on whether the tone is flat, rising, dipping or falling). Findings from tonal language speakers have been inconsistent. Some revealed no significant bimodal benefit on lexical tone recognition either in quiet or in noise [36]. Some reported a significant bimodal benefit for lexical tone recognition in noise but not in quiet [32,35]. Others showed a significant bimodal benefit in quiet for tonal perception tasks that depend more on pitch cues [33,34]. While most of the studies focused on the verification of bimodal benefits in various listening conditions, very few investigated the candidate factors that could account for the individual variability in bimodal benefit. One exception is Chang et al. [31], which reported no significant correlation between the acoustic hearing threshold of the non-implanted ear and the bimodal performance in Mandarin tone recognition. The lack of solid evidence for the underlying contributing factors of the bimodal benefit variability leaves the topic open for further inspection. In particular, there has been little work on young bimodal children who are still under a sensitive period of auditory cortical organization for tonal acquisition.

Several initiatives have been launched to investigate the lexical tone perception in Mandarin children with CIs. Based on differences in pitch height and pitch contour, Mandarin tones are traditionally classified into four categories: Tone 1 (T1) with a high-flat pitch, Tone 2 (T2) with a mid-rising pitch, Tone 3 (T3) with a falling-rising pitch, and Tone 4 (T4) with a high-falling pitch. Prior studies have demonstrated that T1 and T4 are significantly easier to perceive than T2 and T3 for native CI children, with more confusion between the identification of T2 and T3 [21,37]. However, it is still an open question whether additional acoustic hearing from the non-implanted ear with an HA has the potential to enhance the representation of different tone patterns and to alleviate the confusion between T2 and T3. Notably, although large individual variability was found across the studies, age at implantation was negatively correlated with tonal perceptual performance, with earlier implanted children obtaining higher perceptual scores. Age at implantation and duration of CI use have been demonstrated as two major demographic factors contributing to the variability in lexical tone perception in pediatric implantees [38], and the two factors are also recognized as significant predictors of sentence-level intelligibility [39]. Under the scope of neural plasticity, there is a critical period for cochlear implantation, with the auditory plasticity decreasing as age increases, and with the expected cortical organizing as implant experience accumulates [1,40,41]. Thus, the demographic factors need to be considered to explain pediatric participants’ bimodal outcome (including bimodal benefit and benefit magnitude) in both benign and adverse listening conditions.

The primary objective of this study was to investigate the nature of the bimodal benefit for Mandarin tone recognition at both group and individual levels in native kindergarten-aged children who use CI in one ear and HA in the other. Three specific hypotheses were tested: (1) the child participants would show confusion between T2 and T3, which can be alleviated by bimodal hearing; (2) the noisy listening condition would exhibit more robust bimodal benefit than the quiet condition; and (3) the audiometric thresholds and the demographic factors of the participants could account for the bimodal benefit in the preschool bimodal participants. The findings of this study would contribute to our understanding and potential development of age-appropriate intervention programs for aural rehabilitation and speech intervention for the pediatric CI population of tonal language speakers.

## 2. Materials and Methods

### 2.1. Participants

Twenty native Mandarin-speaking preschoolers born with bilateral severe-to-profound hearing loss were recruited from the Shanghai Rehabilitation Center of the Deaf Children. They had no history of psychiatric disorders or brain injuries in their medical history. All the child participants were implanted unilaterally and fitted with a contralateral HA. They used the CI in conjunction with HA devices for at least 75% of waking hours (c.f., [22,28]) according to their caregivers’ observation. However, 6 were removed from data analysis due to failure in completing all experimental tests. The remaining 14 preschoolers with an age range of 4.7 to 6.6 years old (mean = 5.5 years, SD = 0.5 years; 6 females and 8 males) were included for the current report. Their demographic information along with the CI and HA details are listed in Table 1.

**Figure 1:**
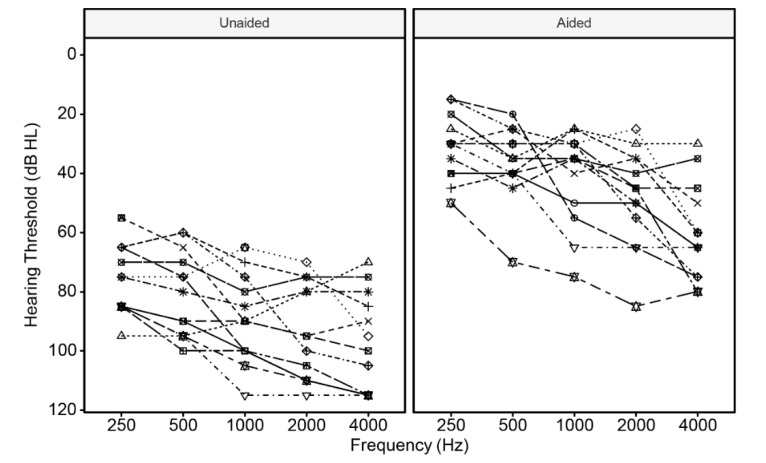
Unaided and aided hearing thresholds for the non-implanted ear of each child participant.

Standard audiometric assessments for pure tones from 125 Hz to 8 kHz were performed to determine the hearing impairment of each child participant. Figure 1 and Supplementary Table S1 show the participants’ unaided and aided hearing thresholds in the non-implanted ear at each frequency. The PTA was calculated for each participant based on the average of hearing thresholds at three low frequencies, 125, 250, and 500 Hz, in the better ear (i.e., the non-implanted ear for the current participants). This approach was adopted in previous studies to examine the relationship between audiometric thresholds and pitch-related speech and music perception in bimodal subjects [42]. In addition, we tested nonverbal intelligence with the Hiskey–Nebraska test of learning aptitude (HNTLA) [43]. All participants scored significantly higher than the passing criteria of 84 for normal nonverbal intelligence. This study was approved by the Ethics Committee of School of Foreign Languages, Shanghai Jiao Tong University. Informed consent was received from the caregiver of each child participant.

### 2.2. Stimuli

**Figure 2:**
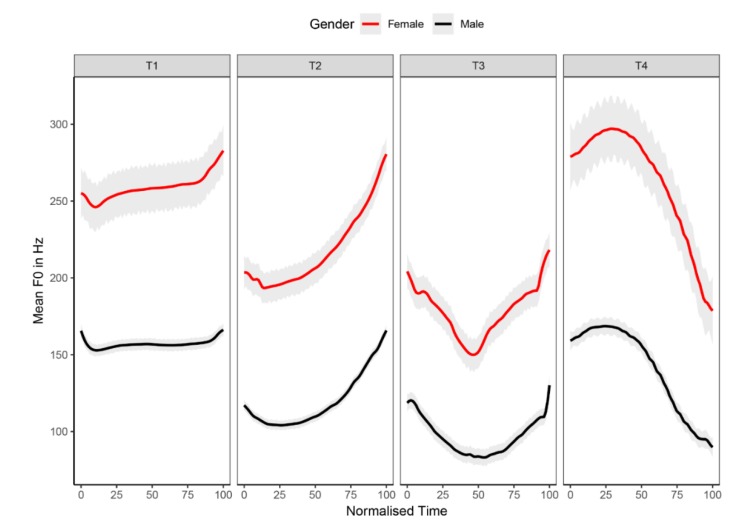
Pitch contours of the lexical tone recognition materials. Grey shades indicate standard error.

Stimuli in the speech recognition task were the four Mandarin tones naturally produced with the /i/ syllable. They were recorded from four native Mandarin-speaking adults (2 males and 2 females) in a double-walled, sound-attenuated booth. Each speaker was required to produce each tone 10 times. The speech samples were recorded at a sampling rate of 44.1 kHz (16 bit). An experienced phonetician selected five speech samples for each tone per speaker, resulting in 80 test stimuli in total. All stimuli were normalized with equal average root-mean-square (RMS) intensity level. The average duration of the T1, T2, T3, and T4 stimuli are 537, 532, 614, and 374 milliseconds (ms), respectively. The pitch contours of the four tones were extracted from the test stimuli using Praat [44], which are depicted in Figure 2 based on speaker genders. The average pitch ranges of T1, T2, T3, and T4 stimuli are 20, 62, 50, and 82 Hz for the male speakers, respectively. By contrast, the accurate pitch ranges are 45, 90, 74, and 122 Hz for the female speakers, respectively, for the four tone types (see Supplementary Table S2 for detailed information).

The lexical tone recognition test was performed in both quiet and noisy listening conditions. To this end, the speech spectrum-shaped noise (SSN) was used as a masker to corrupt the test stimuli at the signal-to-noise ratio (SNR) level of +5 dB. The SSN was generated by passing white noise through a finite impulse response filter derived from the average spectrum of all the speech samples (c.f., [45]). Our pilot test with four SNR levels (i.e., +10, +5, +3, and 0 dB) in five subjects indicated that the + 5dB SNR would be appropriate to avoid both ceiling and floor effects. Previous research had also used the SNR of +5 dB in word and sentence recognition tests for CI participants (e.g., [22,32,46]).

### 2.3. Procedures

All child participants performed the test using their own CI/HA devices and daily settings for volume and sensitivity in a sound-treated therapy room. The preschoolers’ lexical tone recognition was examined in two device conditions and two listening conditions. The order of the two device conditions was randomized and counterbalanced across all participants. In order to reduce the practice and fatigue effects, the measure under different device conditions was performed on two lab visits with a one-week separation.

The test was implemented with E-Prime 2.0 on a Windows-based laptop. The auditory stimuli were delivered in the free field via a loudspeaker (JBL CM220) which was placed approximately 1.2 m from the front of the listener. The stimuli were calibrated to be at a sound level of approximately 65 dB SPL to the location of the child participant’s head. Before the test session, a brief instructional session with practice was provided to ensure that all participants could follow the requirements of the task. The child participants were instructed to perform sound-picture matching with each picture representing the pitch contour of the corresponding tone, from the four options for the four Mandarin tones presented on the laptop computer screen. The four pictures of driving cars are popular materials for teaching the four Chinese lexical tone categories in formal school settings, with a car driving on a level road indicating T1, a car driving on a road with rising slope indicating T2, a car driving on a road with falling and rising slopes indicating T3, and a car driving on a road with a falling slope indicating T4. The experimenter presented each tone with the corresponding picture to explain the relationship for the sound-picture matching to the participant. A practice block was then offered with trial-by-trial feedback. To avoid carryover, the stimuli for the demonstration and practice were not included for the following test. Only after the participant consecutively succeeded in matching the four tone types with the correct pictures in a random order, would she/he be allowed to proceed to the test.

The testing session adopted a four-alternative, forced-choice (4 AFC) paradigm for each stimulus. The preschoolers were required to pay attention to the presented stimulus and encouraged to imitate the sound while pointing to the correct picture. If the vocal response contradicted with their picture pointing, they would be required to clarify their choice. This stipulation allowed the experimenter to double check the participants’ intended response. The child participants were encouraged to make a guess when they were uncertain about the choice. Their trial-by-trial responses were logged by the experimenter via pressing the corresponding keys on the keyboard. The total of 80 tonal stimuli were randomly presented to each child within two blocks. Both tone types and speakers were balanced across the two blocks. Participants had a two-minute break between blocks.

### 2.4. Data Analysis

#### 2.4.1. Accuracy Score and Confusion Matrix

The percentage of accuracy scores and confusion matrices of the four tones were computed for each participant in the two device conditions and the two listening conditions. The percentage score of tone recognition was transformed into a rationalized arcsine unit (RAU) score [47] for statistical analysis. The RAU score was widely used in speech intelligibility tasks to reduce saturation effect and restore homoscedasticity [48].

All statistical analyses were performed with the open source R platform (Version 3.6.0). Linear mixed-effects (LME) models were constructed to evaluate the accuracy scores of lexical tone recognition in quiet and noise for the bimodal participants. The package of lme4 [49] was used to create the LME models. As for fixed effects, tone type (i.e., T1, T2, T3, and T4), device condition (i.e., CI alone and CI + HA), listening condition (i.e., quiet and noise), and all possible interactions were treated as fixed factors. In addition, speaker gender was treated as a control factor, since the intrinsic differences in voice pitch between male and female speakers (see Figure 2) could potentially affect the preschool participants’ lexical tone recognition. The participant was treated as a random effects variable. The full model with all fixed factors was compared with a simplified model that excluded a fixed factor in question using the ANOVA function in lmerTest package [50]. The Akaike information criterion (AIC) was computed to check whether the fixed factor in question could contribute to an improved fit for the constructed model. Eventually, the model with the lowest AIC was selected to estimate the significance of the fixed factors, with α setting at 0.05. In addition, the ANOVA function was used to obtain *F-* and *p*-values of the significant fixed factors, which uses the Satterthwaite approximation for the degree of freedom. Post-hoc pairwise comparisons were performed for the significant fixed factors using the lsmeans package [51] with the Bonferroni adjustment to obtain *t*-ratios, and *p*-values.

Beyond the accuracy scores for tone recognition, we further analysed the error patterns for each device condition in the two listening conditions. Specifically, in order to investigate the confusion between T2 and T3 across the child participants, the error scores with T2 misidentified as T3 and T3 misidentified as T2 were estimated for each participant in each device condition and listening condition. An additional LME model was created to examine the significance of tone type (i.e., T2 and T3), device condition, and listening condition on the misidentified error scores, using by-participant random effects.

#### 2.4.2. Normalized Bimodal Benefit Score

Bimodal benefit is traditionally calculated as the difference in perceptual performance between the CI + HA device condition and CI alone condition. One shortcoming of this absolute calculation, as specified by Zhang et al. [30], is the failure of taking into account the limited improvement room when the participant obtained a high level of perceptual performance in CI alone condition. For example, an improvement of 10% absolute score would be different for a participant obtained from 60% accuracy in CI alone condition to 70% accuracy in CI + HA condition, relative to another participant who improved from 90% to 100% ceiling level. In the first case with a starting score of 60%, there is 40% of room for improvement. In the second case with a 90% starting score, however, there is only 10% room to reach ceiling-level performance. To handle such inter-subject differences, the normalized benefit score was calculated according to the formula (i.e., 100 × ((Bimodal - CI alone) / (100% - CI alone))) as defined specifically in Zhang et al. [30]. In addition, for child participants who achieved lower accuracy score in bimodal condition than in CI alone condition, the formula was reworked (i.e., 100 × ((Bimodal - CI alone) / (CI alone))) to describe the potential drop in lexical tone recognition with an additional HA (c.f., [30]). The normalized formula could control the different performances in CI alone condition into a comparable level across individuals, which contributes to an indexed score ranging from -100 (for extreme cases drop from 100% in CI alone condition to 0% in bimodal condition) to +100 (for extreme cases improve from 0% in CI alone condition to 100% in bimodal condition). In the field of speech and hearing sciences, this normalized equation has been widely adopted in recent studies to evaluate individual’s bimodal benefits in speech intelligibility tasks (e.g., [46,52,53,54,55,56,57]).

#### 2.4.3. Regression Models for Delineating Relationships

The hypothesized predictors for tonal recognition performance consisted of audiometric thresholds and demographic factors. The evaluations of perceptual performance of lexical tones in the current report included (1) lexical tone recognition in CI alone condition, (2) lexical tone recognition in CI + HA condition, and (3) normalized benefit score for lexical tone recognition. Audiometric thresholds included (1) three-low-frequency PTA, (2) five-frequencies unaided hearing threshold, and (3) five-frequencies aided hearing threshold. The key demographic factors were (1) chronological age, (2) implanted age, (3) duration of CI use, and (4) duration of bimodal use. Multivariate regression models were constructed in R to assess the significant variables that contributed to the child participants’ lexical tone performance and the potential bimodal benefit. Separate models were developed for the three estimates of perceptual performance with the audiometric variables and demographic variables added as fixed effects. *F*-statistics, and *p*-values for the fixed effects were assessed with significance level α = 0.05.

## 3. Results

### 3.1. Lexical Tone Recognition

**Figure 3:**
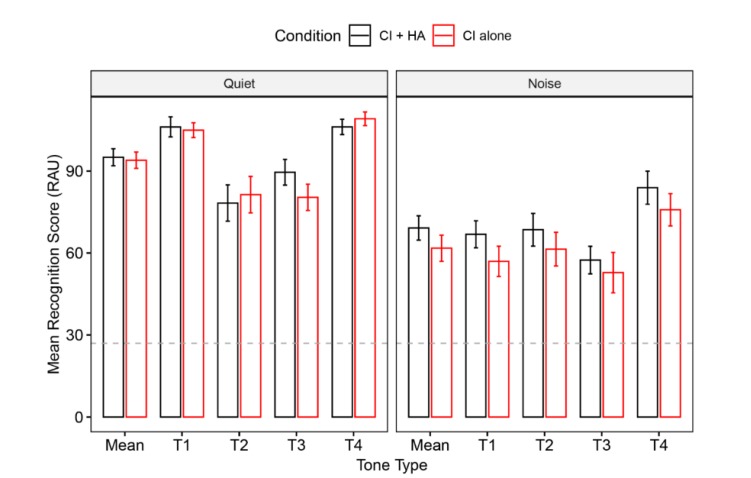
Mean recognition scores in rationalized arcsine unit (RAU) of each tone type in quiet and noise. The dash lines indicate the chance level. Error bars represent the standard errors across all participants.

Group mean accuracy data for the four lexical tones are shown in Figure 3, contrasting performance for the two device conditions in quiet and in noise. The average RAU scores of tonal identification for CI alone and CI + HA device conditions were, respectively, 93.95 (*SD* = 11.09) and 95.04 (*SD* = 11.64) in quiet, and 61.76 (*SD* = 17.91) and 69.17 (*SD* = 16.62) in noise. In quiet, both T1 and T4 obtained nearly perfect scores whereas T2 was mainly misidentified as T3 (accounting for 88.54% of all errors), and T3 was mainly misidentified as T2 (accounting for 87.28% of all errors). In noise, T4 was identified with the fewest errors, and was relatively evenly misidentified as the other three tones. However, T1 was mainly misidentified as T2 (accounting for 49.76% of errors), T2 was mainly misidentified as T3 (accounting for 48.21% of errors), and T3 was mainly misidentified as T2 (accounting for 56.17% of errors). The results demonstrated the confusion between T2 and T3 identification both in quiet and in noise. See Supplementary Table S3 and Table S4 for detailed information of the confusion matrices of the four tones in quiet and noise.

Statistical analysis for the accuracy data revealed a significant main effect of device condition (*F* = 5.96, *p* = 0.02). Compared with CI alone, lexical tones were overall significantly better recognized with bimodal stimulation (*t* = 2.41, *p* = 0.03). The significant main effect of tone type (*F* = 12.99, *p* < 0.001) was also revealed, with the identification of T4 significantly better than all other three tones (all *t*s > 3.19, *p*s < 0.05). Moreover, there were also significant interaction effects of device condition by listening condition (*F* = 4.14, *p* = 0.04). Further post-hoc comparisons in quiet revealed no significant difference on lexical tone recognition between the two device conditions, with only T3 showing marginally better CI + HA performance than with CI alone (*t* = 1.85, *p* = 0.06). By contrast, in noisy listening condition, the perceptual performance of lexical tones was significantly better in bimodal hearing than in CI alone condition (*t* = 3.13, *p* = 0.003).

Additional LME models for error analysis found a significant three-way interaction of tone type by listening condition by device condition (*F* = 4.64, *p* = 0.03). Post-hoc pairwise comparisons with Bonferroni adjustment showed that T3 was less misidentified as T2 for CI + HA condition relative to CI alone condition in quiet (*t* = -2.06, *p* = 0.04), but not in noise (*p* = 0.97).

**Figure 4:**
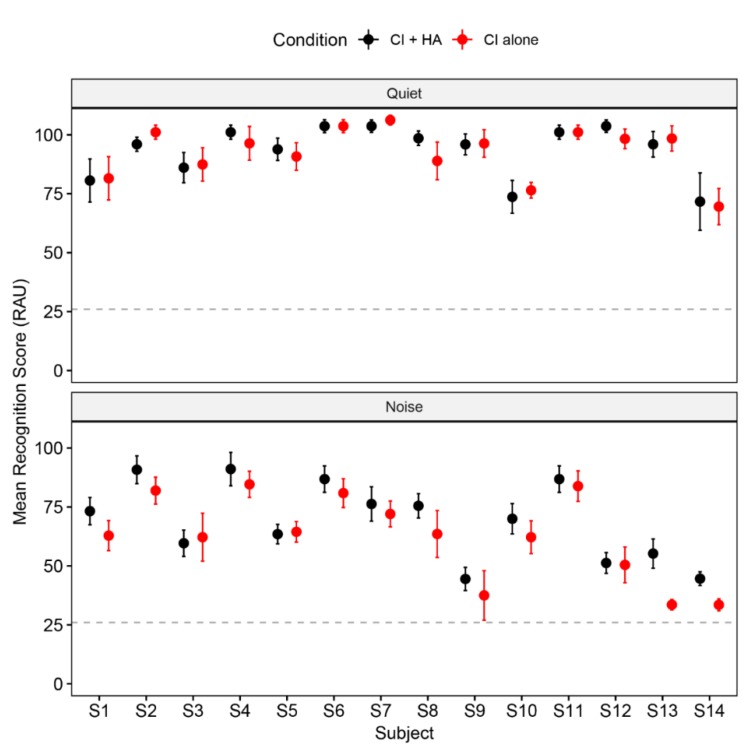
Mean RAU scores of natural tone recognition for each participant in quiet and noise. Dash lines indicate the chance level. Error bars represent the standard errors across four tone types.

The individual listeners’ results are displayed in Figure 4. Paired-samples T tests were conducted to compare the perceptual performance of each individual between CI alone and CI + HA device conditions. In quiet, no subject showed a significant difference between the two device conditions (all *p*s > 0.08). However, in noise, a significant bimodal benefit was revealed for S13 (*t* = 3.69, *p* = 0.005), and S14 (*t* = 4.1, *p* = 0.003).

### 3.2. Normalized Bimodal Benefit

**Figure 5:**
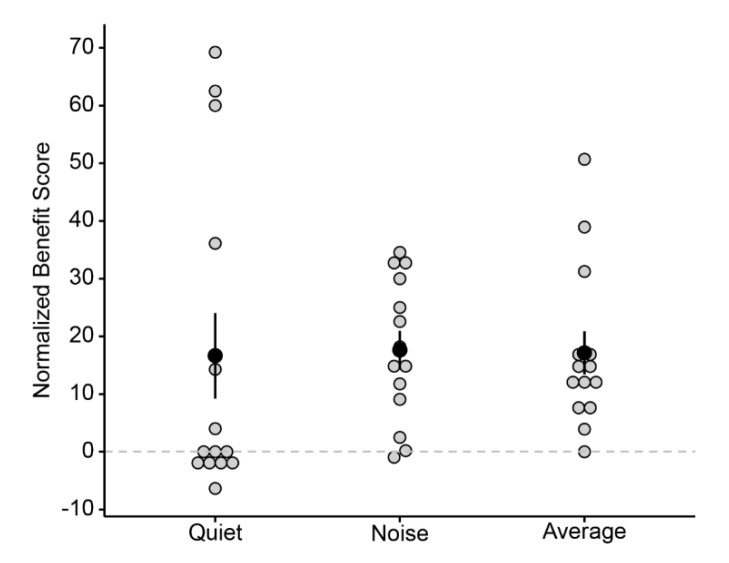
Normalized benefit score received from an additional HA in the non-implanted ear for lexical tone recognition in quiet (left column), in noise (middle column), and the average across the two listening conditions (right column).

To account for individual differences in performance, the normalized benefit score compares the two device conditions and computes relative gain in the bimodal condition over the CI alone condition for each individual. On average, the normalized benefit score on lexical tone recognition was +16.64 (ranging from -6.35 to +69.23) in quiet and +17.64 (ranging from -1.89 to +34.55) in noise. Five children had negative scores in quiet condition, suggesting the lack of bimodal benefit (*p* = 0.42, exact binomial test). In comparison, 12 children showed positive scores in the noisy condition, indicating the tangible bimodal benefits (*p* < 0.01, exact binomial test). The mean normalized benefit score was calculated as the average score of the two listening conditions for each participant. The average score was +17.14 on group level, ranging from 0 to +50.69. The normalized benefit scores for each child participant in quiet and noise are shown in Figure 5.

### 3.3. Regression Analysis Results

For the CI alone condition, the linear regression models revealed that CI duration was marginally associated with lexical tone recognition across listening conditions (*F* = 3.65, *p* = 0.08). Specifically, CI duration was a significant predictor for lexical tone recognition in noise (*F* = 4.89, *p* = 0.047), with increased CI use contributing to a higher accuracy score of tonal recognition in noise, but not in the quiet condition (*p* = 0.45). Similar results were obtained for the CI + HA condition. CI duration was significantly correlated with lexical tone recognition in noise (*F* = 5.59, *p* = 0.036), but not in quiet (*p* = 0.4).

The duration of bimodal use was a significant predictor for the bimodal benefit in lexical tone recognition across the two listening conditions (*F* = 5.73, *p* = 0.034). Overall, the normalized benefit scores improved as a function of increase in the combined use of CI and HA. Moreover, the PTA at three low frequencies in the non-implanted ear was significantly correlated with bimodal benefit in quiet (*F* = 8.09, *p* = 0.015), with a lower PTA resulting in a higher normalized benefit score. A visual inspection of residual plots revealed that the residuals were normally distributed without any obvious deviations from homoscedasticity for each model.

## 4. Discussion

This study investigated the bimodal benefit for Mandarin tone recognition in native kindergarten-aged children who used a CI in one ear and an HA in the opposite ear. Both quiet and noisy listening conditions were tested. The main results were in line with the hypotheses. The additional HA in the non-implanted ear helped alleviate the recognition confusion between T2 and T3 in quiet. Meanwhile, significant bimodal benefits were found for tone recognition in noise. Furthermore, PTA at three low frequencies in the non-implanted ear and the duration of bimodal use were two significant predictors for the potential bimodal benefit in lexical tone recognition.

### 4.1. Bimodal Benefits for Lexical Tone Recognition

Significant bimodal benefits for lexical tone recognition were shown in noisy listening condition, and only limited benefits were exhibited in quiet with additional acoustic information from the contralateral HA alleviating the confusion between T2 and T3. The findings were consistent with previous studies on adult bimodal listeners [32,35], which revealed tangible bimodal benefits in tonal identification in noise but not in quiet.

The error pattern analysis in this study revealed that T2 and T3 were mainly misidentified as each other. The confusion between T2 and T3 was in line with prior reports that examined pediatric Mandarin-speaking CI users’ lexical tone perception in quiet [21,37]. One known source for the confusion between Mandarin T2 and T3 is the acoustic similarity of the two tones [58]. As illustrated in Figure 2, T2 and T3 in isolated speech both have a rising component, and the reliable contrastive feature to discern T3 from T2 is the timing of the inflection point that turns the pitch contour from falling to rising. Given the device-related limitations of CIs in encoding pitch cues, it is reasonable to expect that an additional HA in the non-implanted ear could complement the pitch deficits and reflect the subtle differences between the two tones. Additionally, the enriched phonetic signals and the enhanced tonal distinctiveness could serve as the physical agents of neural plasticity [59], which could be conducive to auditory plasticity for acquiring distinct tonal categories. Taken together, bimodal hearing has the potential to alleviate the confusion between T2 and T3, with better tonal representations retrieved through the bimodal stimulation. Our results from the quiet listening condition provided modest support for the hypothesis. The accuracy score analysis revealed a marginally significant improvement (*p* = 0.06) for T3 identification in bimodal condition over the CI alone condition. Meanwhile, the error score analysis showed a significant decrease (*p* = 0.04) in misidentifying T3 as T2 when an additional HA was used in the non-implanted ear.

One apparent effect of noise on tone recognition in the present study was the significant drop of accuracy across all tone types, which enlarged the room for potential observable bimodal benefits. The accuracy results confirmed a significant bimodal benefit for the lexical tone recognition in noise at the group level. Individual-level examination revealed the majority of the preschoolers (12 out of 14) gained evident bimodal benefit, and two showed significant improvement for bimodal condition over CI alone condition (see Figure 4 and Figure 5).

Which binaural mechanisms may underlie the bimodal benefit in the noisy listening condition? One plausible account is an improved pitch representation from the bilateral bimodal hearing [29,60,61]. Combining the degraded pitch information from the electric stimulation with the complementary low-frequency cue from the acoustic stimulation provides the bimodal listeners with better pitch representations. With the enhanced pitch representations, the child participants should presumably be capable of segregating target tones from SSN maskers, which could eventually contribute to the improved lexical tone recognition in noise with bimodal hearing. An alternative explanation is that ‘glimpsing cues’ may also play an important role in the bimodal benefit in noise [62,63,64]. According to Cooke [65], glimpses are spectral-temporal regions that are least affected by the background noise, and listeners can take advantages of glimpses in perceiving noisy speech. The spectral-temporal region refined by the low-frequency phonetic information from the additional HA might constitute useful glimpsing cues for the bimodal participants to fuse pieces of critical message as acoustic landmarks to ‘bootstrap’ lexical tone identification. In addition, from the perspective of neuroplasticity, bilateral bimodal hearing could prevent aural preference for the implanted ear and promote the cortical integration of input from bilateral auditory pathways [66,67]. Recently, Polonenko et al. [67,68] demonstrated that children with asymmetric hearing loss, who received bimodal input with limited delay (i.e., restricting the duration of unilateral hearing), could reverse abnormal cortical aural preference and recover typical bilateral representations from both ears. Therefore, bimodal hearing is expected to improve speech detection in the noisy condition by providing bilateral representations that are retrieved from sound input from both ears.

### 4.2. Contributing Variables to Bimodal Benefits

Our data showed that both low-frequency PTA in the non-implanted ear and the duration of bimodal configuration for the participants were significant predictors for the potential bimodal benefit in their Mandarin tone recognition. The association between low-frequency PTA and bimodal benefit has been previously reported [30]. In particular, Zhang et al. [30] examined the relationship between the audiometric threshold (≤ 750 Hz) and bimodal benefit with Pearson correlation analyses. Our linear regression analysis incorporated fixed-effect and random-effect variables to better account for between-subject baseline differences and correlational structure among multiple predictor variables [69]. The results confirmed that the bimodal benefit in lexical tone recognition could be predicted by the low-frequency PTA in the non-implanted ear. The findings were compatible with a recent electroencephalography investigation that revealed bimodal hearing might be more effective in restoring typical cortical organization in children with sufficient acoustic hearing in the non-implanted ear [67]. 

Furthermore, one novel finding from our multivariate regression analysis is that the duration of bimodal device use is a significant contributor to the bimodal benefit in the child participants. Although previous studies have examined whether the demographic factors are able to predict CI children’s lexical tone perception [38], no prior studies have reported whether age at bimodal configuration and duration of bimodal hearing are predictive of the bimodal benefit in their tonal performance. Given the critical period of auditory neuronal development and the significance of language exposure for the neural plasticity in speech learning [1,59], it is reasonable to postulate that the early bimodal adoption and the accumulating bimodal experience might contribute to the degree of bimodal benefit. Actually, our data provided the first evidence that the longer use of bimodal configuration might contribute to the development of the binaural ability to integrate acoustic and electric stimulation in the different ears, which could lead to the enhancement of bimodal benefit.

However, it should be noted that some studies failed to show consistent results. For example, Pearson correlation analyses by Chang et al. [31] did not show any significant correlations between bimodal benefit in Mandarin tone recognition and audiometric thresholds in the non-implanted ear or demographic factors of the participants. In addition to differences in statistical modelling, one noticeable difference between Chang et al.’s study and the current report lies in the methodology in calculating bimodal benefit. Chang et al.’s measure had a possible range of 0 to 100 whereas our index score permitted a wider range of −100 to 100.

### 4.3. Limitations and Implications

One main limitation of the present study is the adoption of only one SSN masker with a fixed SNR of 5 dB. The SSN is believed to introduce mostly energetic masking, whereas other types of maskers such as competing speech can induce both energetic and informational masking [70]. It is of great interest to investigate the effect of masking types in the recognition of Mandarin tones for native young children with congenital hearing loss. Meanwhile, our findings revealed considerable variability in lexical tone recognition with 5 dB SSN masker in the full range of near-ceiling level performance in some participants and floor level outcome in some others (see Figure 4). Both ceiling and floor effects limit the degree of bimodal benefit. Therefore, an individualized SNR with moderate speech reception threshold (e.g., [42,46,61]) would be an alternative approach in future investigations on the bimodal benefit for lexical tone perception.

The second limitation lies in the relatively high participant heterogeneity and small subject sample, with only six girls and eight boys. This confined our foray into examining the potential gender differences in the bimodal benefits for lexical tone recognition. To obtain robust statistical results, a much larger sample size with better-controlled demographic characteristics is desirable. In addition, although all child participants in our study were reported to be full-time users of CI devices and over 75% waking-hour users of HA devices, it should be acknowledged that the reports were based on their caregivers’ estimation. Wearing time of the devices has a direct impact on the perceptual performance of the bimodal listeners [46], and not all bimodal recipients adapt well to their contralateral HA [71]. Therefore, further research is needed with proper measures of actual device wearing time and the integration ability of bimodal stimulation among the listeners.

The third limitation is in regards to the proper method for assessing bimodal benefits with the baseline performance taken into account. Although recent years have witnessed a growing trend of using the normalized equation in estimating the benefit magnitude of each individual [30,46,52,53,54,55,56,57], this calculation method could run the risk of artificially inflating the magnitude of the bimodal benefit when the baseline performance for comparison is close to the ceiling level. Thus due caution is necessary to interpret the bimodal benefits as reported in our study and others. It would be important to develop an optimal protocol with a standardized scoring method to take into account the starting level of performance in the CI alone condition and avoid the potential inflation for the benefit magnitude.

Due to time consideration for feasible test protocols with children in a laboratory setting, the current study was restricted to testing the lexical tones in the /i/ syllable in quiet and in noise. Thus, further validation efforts are needed to test the generalizability of the bimodal benefits for lexical tone perception with other types of stimuli including other syllable, words and sentences as the listener would encounter in the real world.

Despite these inherent limitations, the present findings have practical implications for intervention. Overall, consistent with the majority of studies on non-tonal language speakers, our findings lend support to the clinical trial of fitting a contralateral HA in the non-implanted ear for the potential bimodal benefit in lexical tone perception for kindergarten-aged children with CIs. As a tonal language, Mandarin poses a great reliance on the pitch information for speech perception. Given the fact that an HA may provide more fine-grained low-frequency signals than a CI to improve the pitch representation, bimodal configuration can be a preferable alternative to a second CI basing on the residual acoustic hearing of the recipient’s non-implanted ear. Therefore, the potential bimodal benefit should be taken into consideration in the clinical audiologic assessment for bilateral CIs in Mandarin-speaking candidates. A second CI can be recommended in the absence of bimodal benefit in quiet as well as in noise, which can provide empirical support for evidenced-based clinical practice.

The auditory rehabilitation and cochlear implantation programs in China have experienced considerable advances in the past decades. The CI candidacy criteria have extended to include individuals with a severe hearing loss. As a result, more CI candidates have aidable acoustic hearing in the non-implanted ear. The residual acoustic hearing, although it has limited use on its own, is demonstrated as providing a substantial benefit in speech perception when combined with the CI stimulation (e.g., [22,62]). The residual acoustic hearing should be taken into consideration for these individuals while setting up intervention regimens. Training studies could be designed with computer-based intervention [6,72] to manipulate the speech input parameters for maximizing bimodal learning.

## 5. Conclusions

Findings of the present study confirmed a confusion pattern for T2 and T3 in Mandarin preschoolers using a CI and an HA in opposite ears. The additional low-frequency acoustic information from the HA in the non-implanted ear can alleviate the confusion between T2 and T3 in quiet, and contribute to an improvement in overall performance of lexical tone recognition in noise. Moreover, audiometric thresholds of PTA at low frequencies (125, 250, and 500 Hz) and bimodal duration were two significant predictors for the varying degrees of bimodal benefit in lexical tone recognition. These results provide evidence for the practice of fitting an HA in the non-implanted ear to take advantage of the potential bimodal benefit to facilitate speech learning in kindergarten-aged children with a unilateral CI. Further research is needed to examine auditory plasticity on an individual basis to gain insights on the contributing factors to the bimodal benefit or its absence in quiet and in noise.

## Figures and Tables

**Table 1:** Demographic information of the participants.

Subject (Sex)	CA (yrs)	CI (ear)	Speech Strategy	HA	Age at CI (yrs)	CI Duration (yrs)	Age at Bimodal (yrs)	Bimodal Duration (yrs)	PTA (dB HL)
S1 (M)	4.9	OPUS2 (R)	FS4-P	Phonak Bolero Q50	0.9	4.0	0.9	4.0	85
S2 (M)	5.9	OPUS2 (R)	FS4-P	Widex C4-FS	0.9	5.0	0.9	5.0	83
S3 (M)	5.6	Naida (R)	HiRes-Optima	Phonak Naida S IX	2.7	2.9	2.7	2.9	78
S4 (M)	5.3	Nucleus6 (L)	ACE	ReSound AL777	2.7	2.6	3.6	1.7	67
S5 (M)	5.7	OPUS2 (R)	FS4-P	Widex C3-FS	1.7	4.0	1.7	4.0	65
S6 (M)	5.6	OPUS2 (R)	FS4-P	Phonak Naida S IX	1.0	4.6	1.0	4.6	88
S7 (M)	4.8	Nucleus6 (R)	ACE	Phonak Naida S IX	1.5	3.3	1.5	3.3	83
S8 (M)	6.6	OPUS2 (R)	FS4-P	Widex C4-FS	1.4	5.2	4.6	2.0	68
S9 (F)	5.6	Nucleus6 (L)	ACE	Phonak Q90 SP	3.3	2.3	3.3	2.3	56
S10 (F)	5.4	Nucleus6 (R)	ACE	Phonak Naida S IX	3.0	2.4	3.7	1.7	92
S11 (F)	6.2	Nucleus5 (R)	ACE	Phonak Naida S IX	1.1	5.1	1.2	5.0	63
S12 (F)	5.5	Nucleus6 (R)	ACE	Phonak SKY Q90-RIC	2.8	2.7	2.8	2.7	60
S13 (F)	5.8	Nucleus5 (L)	ACE	Phonak Q90 UP	3.2	2.6	3.3	2.5	77
S14 (F)	4.7	Freedom (R)	ACE	Phonak Naida S V SP	1.2	3.5	1.2	3.5	83

CA: chronological age; CI: cochlear implant; HA: hearing aid; yrs: years; PTA: unaided three-frequency pure-tone average at 125, 250, and 500Hz for the non-implanted ear.

## Supplementary Materials

The following are available online at https://www.mdpi.com/2076-3425/10/4/238/s1, Table S1: Unaided and aided hearing thresholds for the non-implanted ear of the preschool participants; Table S2: Pitch contour information of each tone type; Table S3: Confusion matrix of lexical tone recognition in quiet; Table S4: Confusion matrix of lexical tone recognition in noise.
