# Supplementary material for: Bimodal Benefits for Lexical Tone Recognition: An Investigation on Mandarin-speaking Preschoolers with a Cochlear Implant and a Contralateral Hearing Aid
Source: Brain Sci. 2020 Apr 17;10(4):238. doi: 10.3390/brainsci10040238 (PMC7226140; doi:10.3390/brainsci10040238)
Supplement: Supplementary file 1 [file brainsci-10-00238-s001.pdf]

Supplementary Materials

Table S1. Unaided and aided hearing thresholds for the non-implanted ear of the preschool participants.

	Unaided thresholds (dB HL)					Aided threshold (dB HL)				
**Subject**	**250Hz**	**500 Hz**	**1 kHz**	**2 kHz**	**4 kHz**	**250Hz**	**500 Hz**	**1 kHz**	**2 kHz**	**4 kHz**
S1	85	90	100	110	>115*	40	40	50	50	65
S2	85	90	90	95	100	40	40	35	45	45
S3	75	80	85	80	80	35	45	35	50	65
S4	65	60	75	100	105	15	25	30	55	75
S5	65	75	100	110	115	15	20	55	65	75
S6	85	95	105	110	>115*	50	70	75	85	80
S7	85	100	100	105	115	30	30	30	45	80
S8	70	70	80	75	75	20	35	35	40	35
S9	55	50	65	105	110	40	30	35	45	60
S10	95	95	90	80	70	25	35	25	30	30
S11	65	60	70	75	85	45	40	25	35	60
S12	55	65	90	95	90	30	25	40	35	50
S13	75	75	65	70	95	30	30	30	25	60
S14	85	95	115	>115*	>115*	30	40	65	65	65

* No response within the audiometer limit of 115 dB HL.

Table S2. Pitch contour information of each tone type.

	Female speakers			Male speakers		
**Tone types**	**Upper (Hz)**	**Lower (Hz)**	**Range (Hz)**	**Upper (Hz)**	**Lower (Hz)**	**Range (Hz)**
Tone 1	285 (25)	240 (20)	45 (18)	171 (16)	151 (6)	20 (15)
Tone 2	281 (25)	191 (17)	90 (15)	166 (11)	104 (3)	62 (9)
Tone 3	221 (16)	147 (12)	74 (19)	130 (9)	81 (10)	50 (12)
Tone 4	300 (41)	178 (10)	122 (37)	170 (12)	87 (14)	82 (17)

Scores outside brackets are average values, whereas inside brackets are standard deviations; Upper: upper bound of pitch contour; Lower: lower bound of pitch contour; Range: the range value of pitch contour.

Table S3. Confusion matrix of lexical tone recognition in quiet.

**Lexical tones**	**Response (%)** Tone 1	Tone 2	Tone 3	Tone 4
Tone 1	**96.8 (96.4)**	1.8 (1.4)	0.7 (0.7)	0.7 (1.4)
Tone 2	2.9 (0.7)	**78.6 (77.8)**	18.6 (20.1)	0 (1.4)
Tone 3	1.1 (1.4)	18.2 (11.2)	**79.6 (86.7)**	1.1 (0.7)
Tone 4	0 (0)	0.4 (1.4)	1.4 (1.4)	**98.2 (97.1)**

Boldface indicates identification accuracies. Scores in brackets indicate performance in CI + HA condition, whereas scores without brackets indicate performance in CI alone condition.

Table S4. Confusion matrix of lexical tone recognition in noise.

**Lexical tones**	**Response (%)**			
	Tone 1	Tone 2	Tone 3	Tone 4
Tone 1	**57.1 (66.8)**	21.4 (16.4)	12.9 (10.7)	8.6 (6.1)
Tone 2	11.1 (11.8)	**61.4 (68.2)**	18.9 (15.0)	8.6 (5.0)
Tone 3	14.3 (13.9)	25.4 (25.0)	**52.9 (57.5)**	7.5 (3.6)
Tone 4	5.4 (6.1)	8.6 (8.6)	10.7 (4.3)	**75.4 (81.1)**

Boldface indicates identification accuracies. Scores in brackets indicate performance in CI + HA condition, whereas scores without brackets indicate performance in CI alone condition.
